# Effect of thoracic paravertebral nerve block on delirium in patients after video-assisted thoracoscopic surgery: a systematic review and meta-analysis of randomized controlled trials

**DOI:** 10.3389/fneur.2024.1347991

**Published:** 2024-04-10

**Authors:** Xuelei Zhou, Wei Mao, Li Zhao, Hongyu Zhu, Linlin Chen, Ying Xie, Linji Li

**Affiliations:** Department of Anesthesiology, The Second Clinical Medical College, North Sichuan Medical College, Nanchong Central Hospital, Nanchong, China

**Keywords:** delirium, thoracic paravertebral nerve block, thoracic surgery thoracoscopy, thoracoscope, postoperative analgesia, meta-analysis

## Abstract

**Background:**

Nerve blocks are widely used in various surgeries to alleviate postoperative pain and promote recovery. However, the impact of nerve block on delirium remains contentious. This study aims to systematically evaluate the influence of Thoracic Paravertebral Nerve Block (TPVB) on the incidence of delirium in patients post Video-Assisted Thoracoscopic Surgery (VATS).

**Methods:**

We conducted a systematic search of PubMed, Embase, Web of Science, Cochrane Library, and Scopus databases in June 2023. The search strategy combined free-text and Medical Subject Headings (MeSH) terms, including perioperative cognitive dysfunction, delirium, postoperative cognitive dysfunction, paravertebral nerve block, thoracic surgery, lung surgery, pulmonary surgery, and esophageal/esophagus surgery. We utilized a random effects model for the analysis and synthesis of effect sizes.

**Results:**

We included a total of 9 RCTs involving 1,123 participants in our study. In VATS, TPVB significantly reduced the incidence of delirium on postoperative day three (log(OR): −0.62, 95% CI [−1.05, −0.18], *p* = 0.01, *I*^2^ = 0.00%) and postoperative day seven (log(OR): −0.94, 95% CI [−1.39, −0.49], *p* < 0.001, *I*^2^ = 0.00%). Additionally, our study indicates the effectiveness of TPVB in postoperative pain relief (*g*: −0.82, 95% CI [−1.15, −0.49], *p* < 0.001, *I*^2^ = 72.60%).

**Conclusion:**

The comprehensive results suggest that in patients undergoing VATS, TPVB significantly reduces the incidence of delirium and notably diminishes pain scores.

**Systematic review registration:**

CRD42023435528. https://www.crd.york.ac.uk/PROSPERO.

## 1 Introduction

For Lung cancer serves as a prominent contributor to cancer-related fatalities across the globe, accounting for nearly a quarter of all cancer-related deaths (1). Despite recent advancements in targeted therapies, immunotherapy, and radiation treatment for lung cancer, early-stage surgical resection remains the predominant curative strategy for the vast majority of patients (2). Video-Assisted Thoracoscopic Surgery (VATS) remains the favored technique for surgical resection in early-stage lung cancer (3). Nonetheless, the rate of postoperative complications in elderly patients who undergo thoracic surgery varies from 12 to 47%, with delirium frequently observed (4). Postoperative delirium is defined as delirium occurring within one week after surgery or before the patient’s discharge from the hospital (whichever occurs earlier), meeting the diagnostic criteria for delirium as outlined in the fifth edition of the Diagnostic and Statistical Manual of Mental Disorders (5). Delirium is categorized into two types based on timing: emergence delirium and postoperative delirium (6, 7). Additionally, delirium is divided into three subtypes based on activity levels: hypoactive delirium, hyperactive delirium, and mixed-type delirium (8, 9).

The presence of delirium has been definitively associated with higher patient mortality rates, reduced comfort during hospital stays, prolonged discharge times, increased hospital expenses, and additional strain on both patients’ families and the healthcare system (10, 11). Recent studies indicate that the implementation of suitable preventive measures can effectively decrease the occurrence of delirium (12), thereby improving patient comfort and the overall quality of healthcare.

Current studies suggest that delirium arises from a combination of various factors, where neuroinflammation plays a pivotal role in both its onset and progression (13, 14). Pain is universally recognized as a leading risk factor for post-surgical delirium (15). Nerve block is believed to have the potential to decrease postoperative pain (16, 17) and diminish the stress and inflammatory responses triggered by surgery (18–21), thus potentially reducing the occurrence of postoperative delirium. However, there is still an ongoing debate regarding whether nerve block can effectively lower the incidence of delirium (22, 23). This study aims to investigate the potential impact of Thoracic Paravertebral Nerve Block (TPVB) on postoperative delirium in patients undergoing VATS.

## 2 Methods

### 2.1 Study design

This study rigorously adhered to the principles outlined in the Preferred Reporting Items for Systematic Reviews and Meta-Analyses (PRISMA) (24) and Assessing the methodological quality of Systematic Reviews (AMSTAR) (25) guidelines. All data incorporated into our study were exclusively derived from published literature, obviating the necessity for ethical review. Furthermore, the study is registered in the International Prospective Register of Systematic Reviews (PROSPERO CRD42023435528).

### 2.2 Data collection

In June 2023, a comprehensive search was carried out across various databases, including PubMed, Embase, Web of Science, Cochrane Library, and Scopus. The retrieval strategy combines a mixture of free-text and MeSH terms, including perioperative neurocognitive disorders, delirium, postoperative cognitive dysfunction, paravertebral nerve block, thoracic surgery, pulmonary surgery, lung surgery, esophageal/esophagus surgery, and others (Supplementary Appendix 1).

### 2.3 Data selection

Two independent researchers conducted a comprehensive and independent assessment and review of the literature, covering titles, abstracts, and full-text articles, to determine the final inclusion of studies in this research. Any disagreements were resolved through discussion, and in cases where a consensus could not be reached, a third researcher intervened to make the final decision.

Inclusion Criteria.

The following criteria were employed for the inclusion of studies:

1. The literature must comprise a randomized controlled trial.
2. The study subjects must be patients undergoing thoracic or pulmonary surgery.
3. Paravertebral nerve block must be administered during the perioperative period.
4. The literature must evaluate the incidence of delirium.
5. Literature from the control group must also be incorporated into the study.

Exclusion Criteria.

The following criteria were applied for the exclusion of studies:

1. Literature classified as case reports.
2. Observational studies.
3. Retrospective cohort studies.
4. Review articles.
5. Trial protocols.
6. Insufficient or unclear data within the literature.
7. Inability to access the full text or contact the authors.

### 2.4 Data extraction and integration

We have developed a data extraction table and conducted a preliminary test. Subsequently, two researchers independently conducted data extraction. Any discrepancies in data extraction were thoroughly discussed. In cases where the two independent researchers were unable to reach a consensus, a third researcher was consulted to make the final decision. The data extraction table comprises the following key elements: author names, publication year, study design, participant age, participant count, type of surgery, anesthesia agent types and dosages, Delirium incidence, and postoperative pain scores. These data were primarily sourced from numerical data presented in tables and figures. We employed the online tool WebPlotDigitizer (Version 4.6; WebPlotDigitizer, A. Rohatgi, Pacifica, CA, United States) to extract data presented in graphical form. We employed the equation proposed by Wan et al. to estimate the mean and standard deviation of data described with medians (interquartile range) (26).

### 2.5 Bias risk assessment and evidence quality grading

We employed the Cochrane Collaboration’s Risk of Bias 2 tool (27) to assess potential bias in the included studies. This comprehensive tool evaluates various aspects, including random sequence generation, allocation concealment, blinding of patients, healthcare providers, data collectors, and outcome assessors, completeness of outcome data, selective outcome reporting, and other potential sources of bias. Each article was then categorized into one of three risk levels: “low,” “some concerns,” or “high.” To evaluate the quality of evidence for each outcome, we applied the Grading of Recommendations, Assessment, Development, and Evaluation (GRADE) methodology (28). This systematic approach allowed us to categorize the quality of evidence as either very low, low, moderate, or high.

### 2.6 Data analysis methods

We carried out data analysis leveraging Stata 17.0 and Review Manager 5.4 software platforms. To gauge the extent of variability among the selected studies, we employed τ^2^ (Tau squared) and *I*^2^ (I-squared) statistical metrics. These statistics were used to gauge and measure the degree of heterogeneity within the gathered data, thereby enabling a more rigorous interpretation of the findings (29). To optimally mitigate potential confounders and to more accurately mirror real-world scenarios, we opted for a random-effects model (30). Notably, when heterogeneity is notably low, outcomes from the random-effects model align with those of the fixed-effects model (30). Consequently, this study uniformly applied a random-effects model for calculating and amalgamating log-odds ratios (log(OR)) and their corresponding 95% confidence intervals (CI) in binary data, as well as in determining Hedges’s *g* (*g*) and their relevant 95% CI in continuous outcomes. This methodology bolsters the accuracy and dependability of our analyses.

In this study, we employed log(OR) and *g* as statistical measures to assess the effect size differences between the experimental group and the control group. Essentially, log(OR) measures the same relationship as the odds ratio(OR), but it provides a more stable and normally distributed estimate, making it more suitable for studies with small sample sizes. On the other hand, g is a standardized measure of mean difference that accounts for the impact of sample size, allowing it to be compared with results from other studies. Compared to the simple standardized mean difference, g adjusts for estimation bias in studies with small samples, thereby offering a more accurate measure of effect size.

To assess and scrutinize publication bias for each assessed outcome, we utilized funnel plots and conducted Egger’s test as part of our analysis (29).

## 3 Results

### 3.1 Inclusion of studies

**Figure 1:**
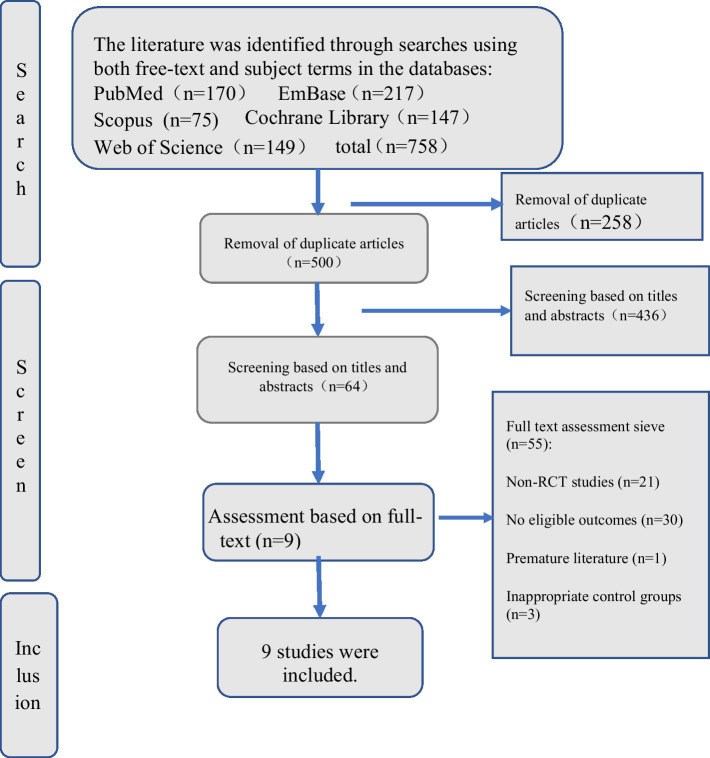
Flow diagram of study selection.

The investigators commenced their study with an initial database search, encompassing PubMed (*n* = 170), EMBASE (*n* = 217), Cochrane Library (*n* = 147), Web of Science (*n* = 149), and Scopus (*n* = 75), culminating in a total of 758 articles obtained. Subsequently, we removed 258 redundant articles. A pair of researchers screened out 436 papers based on their titles and abstracts. Subsequently, 64 articles underwent full-text assessment by the same duo, resulting in a final selection of 9 articles. For a comprehensive selection process, please consult Figure 1.

### 3.2 Study characteristics

In this study, a total of eight studies conducted thoracoscopic lung lobe resection under general anesthesia combined with TPVB (31–38), while another study performed thoracoscopic surgery under general anesthesia combined with TPVB (39). In seven of the studies, the postoperative analgesia regimen employed Patient-Controlled Intravenous Analgesia (PCIA). In the remaining two studies, the pain management approach involved either continuous thoracic epidural analgesia or PCIA. Detailed information on each study can be found in Table 1.

**Table 1:** The characteristics of included studies.

Source	Type of surgery	Type of local anesthetics (Dose)	Measurement of postoperative delirium	Measurement of pain	Control			TPVB		
					Age(years)	Sample size	Postoperative pain management	Age (years)	Sample size	Postoperative pain management
Dongjie et al. (31)	VATS	Ropivacaine (0.375% 20 mL)	Nu-DESC	VAS	69 ± 4	105	PCIA	68 ± 5	103	PCIA
Wei et al. (32)	VATS	lidocaine (1% 5 mL)	3D-CAM	VAS	73.5 ± 7.1	168	PCIA	76.2 ± 6.3	170	TPVB
Zhang et al. (33)	VATS	Ropivacaine (0.5% 30 mL)	NI	VAS	52.13 ± 6.55	23	PCIA	54.32 ± 6.56	22	PCIA
Heng et al. (34)	VATS	Ropivacaine (0.5% 20 mL)	Nu-DESC	VAS	69.7 ± 6.1	64	PCIA	70.3 ± 5.5	64	TPVB
Chen et al. (35)	VATS	Ropivacaine (0.375% 20 mL)	MMSE	VAS	56.46 ± 6.07	37	PCIA	58.81 ± 5.58	36	PCIA
Liu et al. (36)	VATS	Ropivacaine (0.75% 7.5 mL) and lidocaine (2% 2.5 mL)	AFPS	VAS	63.8 ± 7.6	48	PCIA	62.4 ± 7.6	49	PCIA
Zhao et al. (37)	VATS	Ropivacaine (0.5% 100 mL)	MoCA	NI	65 ± 7	35	PCIA	65 ± 6	35	PCIA
Wei et al. (39)	VATS	Ropivacaine (0.2% 1 mg/kg)	PAED	FLACC	4.3 ± 2.3	29	PCIA	5 ± 1.6	29	PCIA
Xie et al. (38)	VATS	Ropivacaine (0.375% 8-15 mL) and lidocaine (2% 3 mL)	MMSE	NI	76.63 ± 4.60	39	PCIA	75.13 ± 5.60	37	PCIA

Age is presented as mean ± standard deviation. TPVB, Thoracic Paravertebral Nerve Block; VATS, Video-Assisted Thoracoscopic Surgery; Nu-DESC, Nursing Delirium Screening Scale; 3D-CAM, 3-Minute Diagnostic Confusion Assessment Method; MMSE, Mini-Mental State Examination; AFPS, Aono’s four-point scale (AFPS) score; MoCA, Montreal Cognitive Assessment; PAED, Pediatric Anesthesia Emergence Delirium scale; VAS, Visual Analog Scale; FLACC, Face, Legs, Activity, Cry, Consolability, PCIA: Patient-Controlled Intravenous Analgesia.

### 3.3 Risk of bias and evidence quality grading

**Figure 2:**
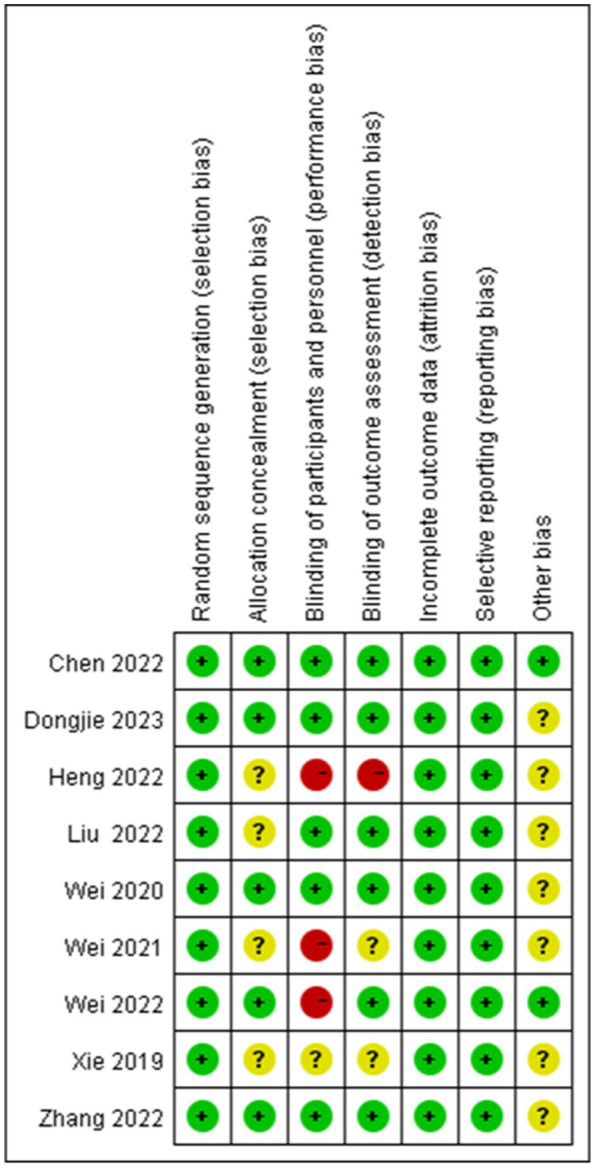
Risk of bias summary: the author’s assessment of the bias risk factors in various studies.

**Figure 3:**
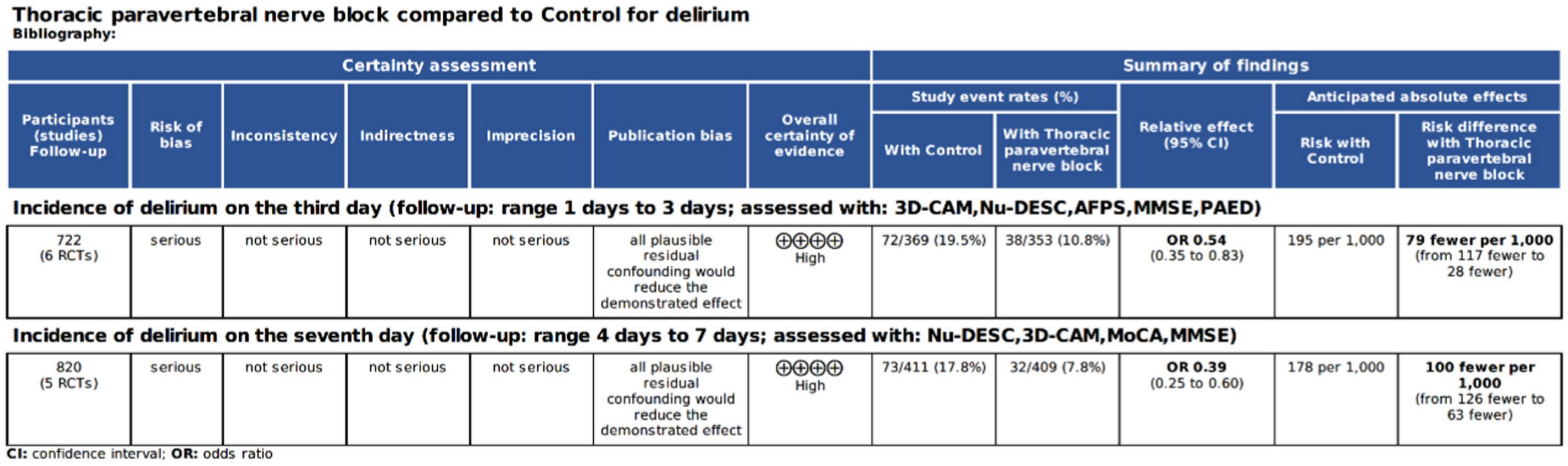
GRADE evidence summary table.

A single study was categorized as posing a low risk of bias (35), whereas seven studies elicited some level of concern regarding bias risk (31–33, 36–39), and another study was flagged for high bias risk (34), as depicted in Figure 2. The quality assessment of the evidence is outlined in Figure 3.

### 3.4 The impact of delirium after surgery within three days

**Figure 4:**
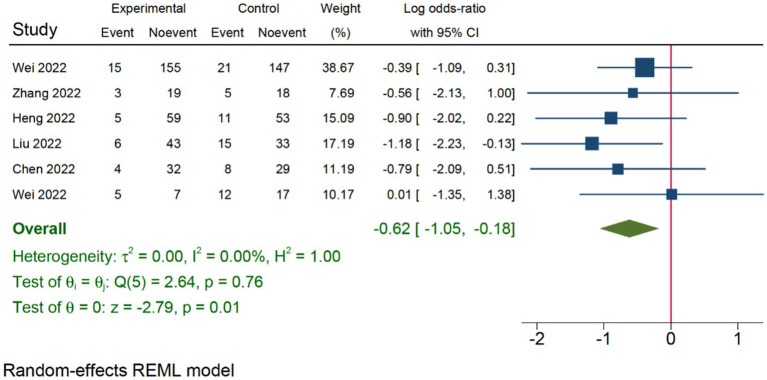
The impact of TPVB on postoperative delirium three days after surgery.

**Figure 5:**
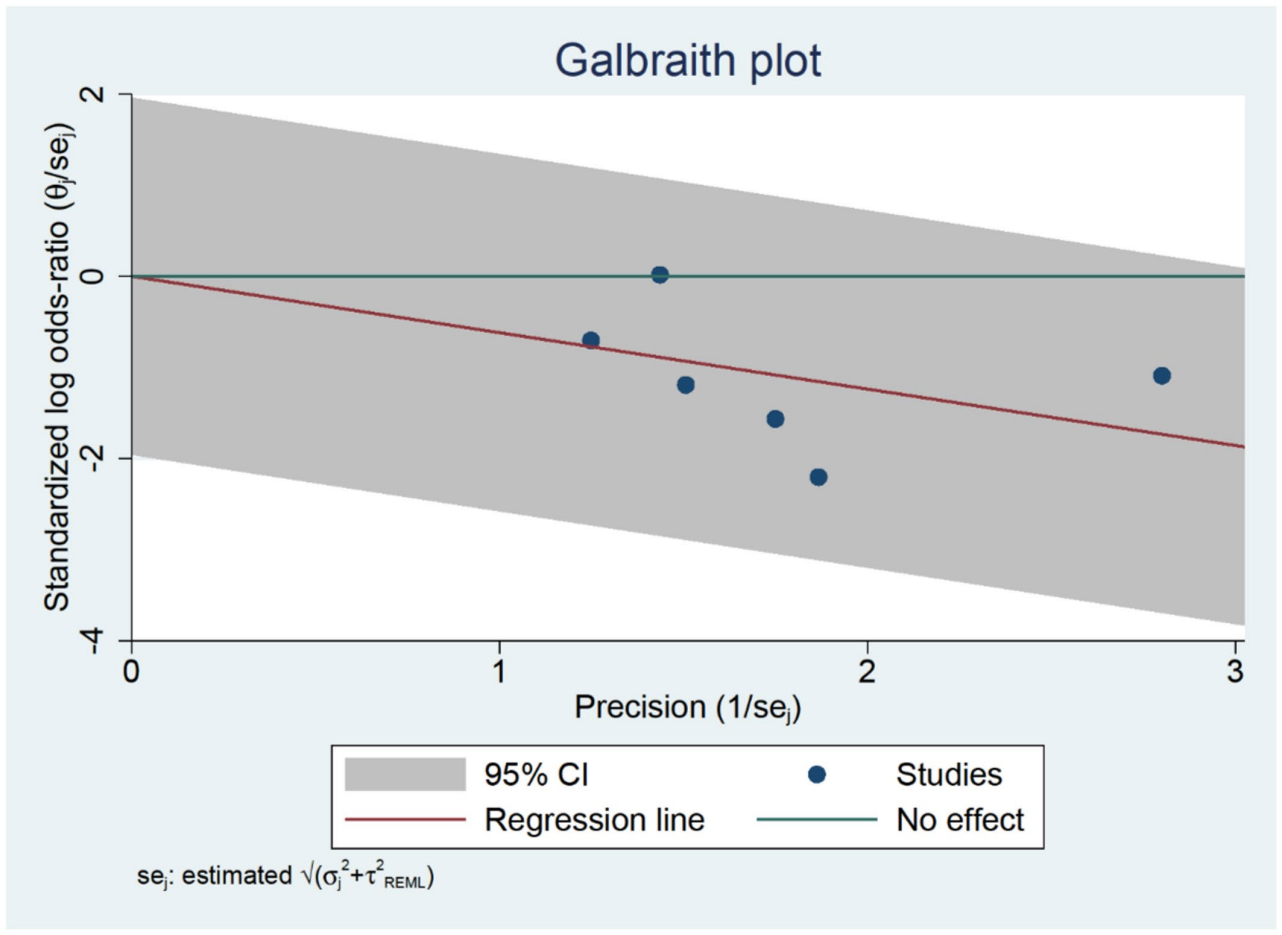
Galbraith plot chart at three days post-surgery.

**Figure 6:**
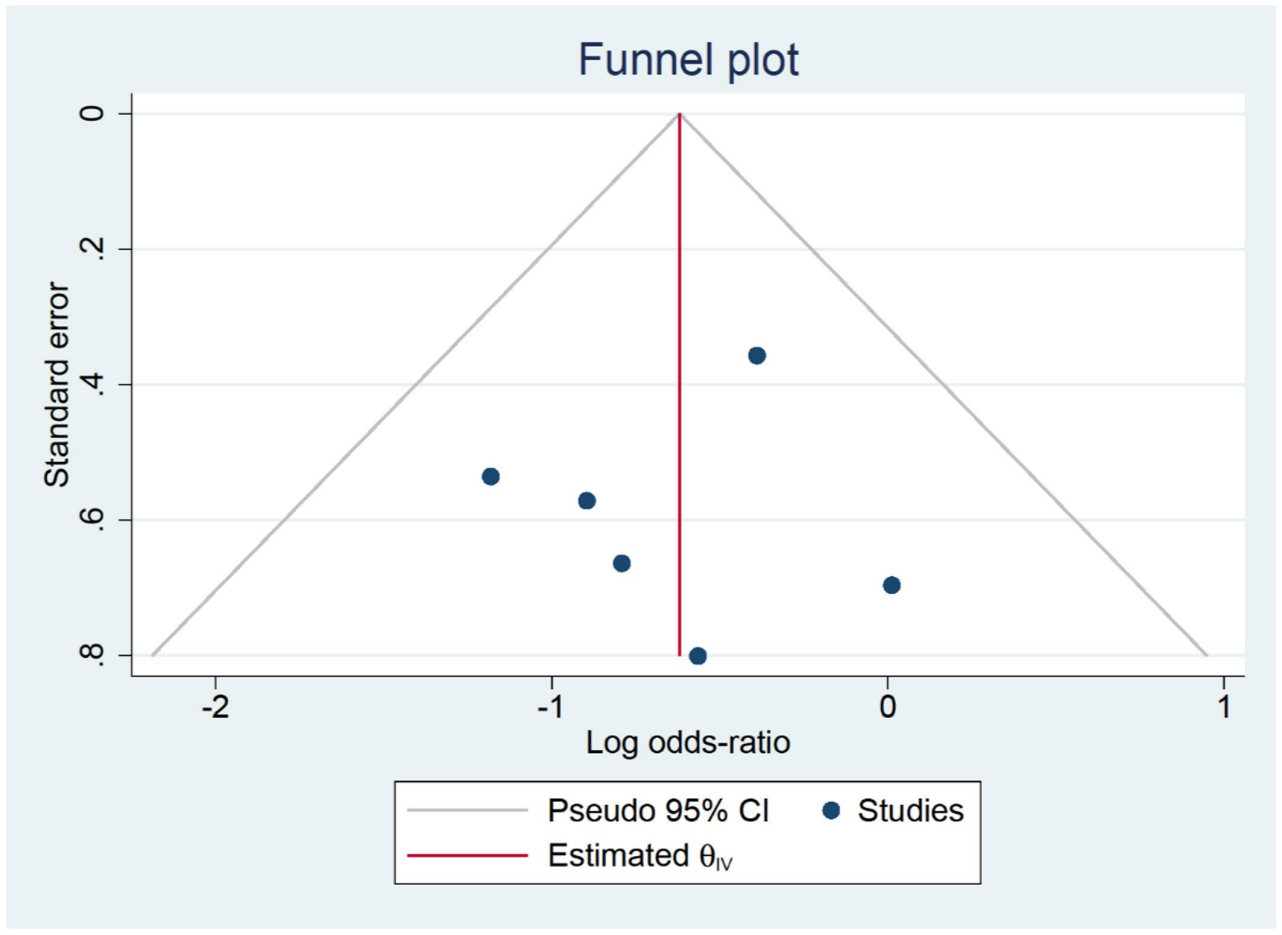
Publication bias funnel plot at three days post-surgery.

Six RCTs studies (32–36, 39) were included in our postoperative three-day delirium analysis. We incorporated the delirium incidence rate (1–3 days) from each study that was closest to the third day. The TPVB group exhibited a lower delirium incidence rate three days post-surgery compared to the control cohort (log(OR): −0.62, 95% CI [−1.05, −0.18], *p* = 0.01, *I*^2^ = 0.00%) (Figure 4). No substantial heterogeneity was identified via Galbraith Plot evaluation (Figure 5). We conducted a publication bias funnel plot (Figure 6) and Egger’s test (*p* = 0.767; Supplementary Appendix 2), both of which did not indicate significant evidence of publication bias.

### 3.5 The impact of delirium after surgery within seven days

**Figure 7:**
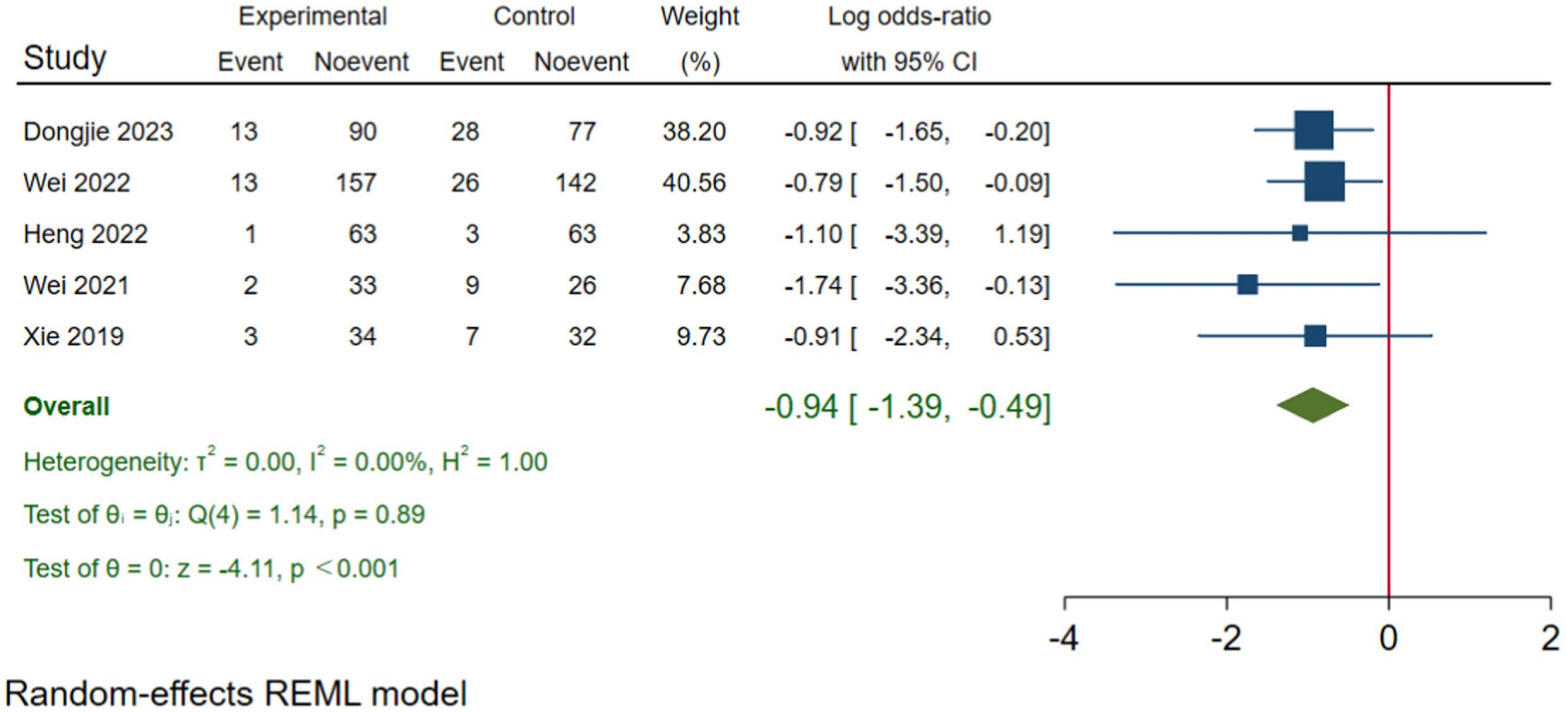
The impact of TPVB on postoperative delirium seven days after surgery.

**Figure 8:**
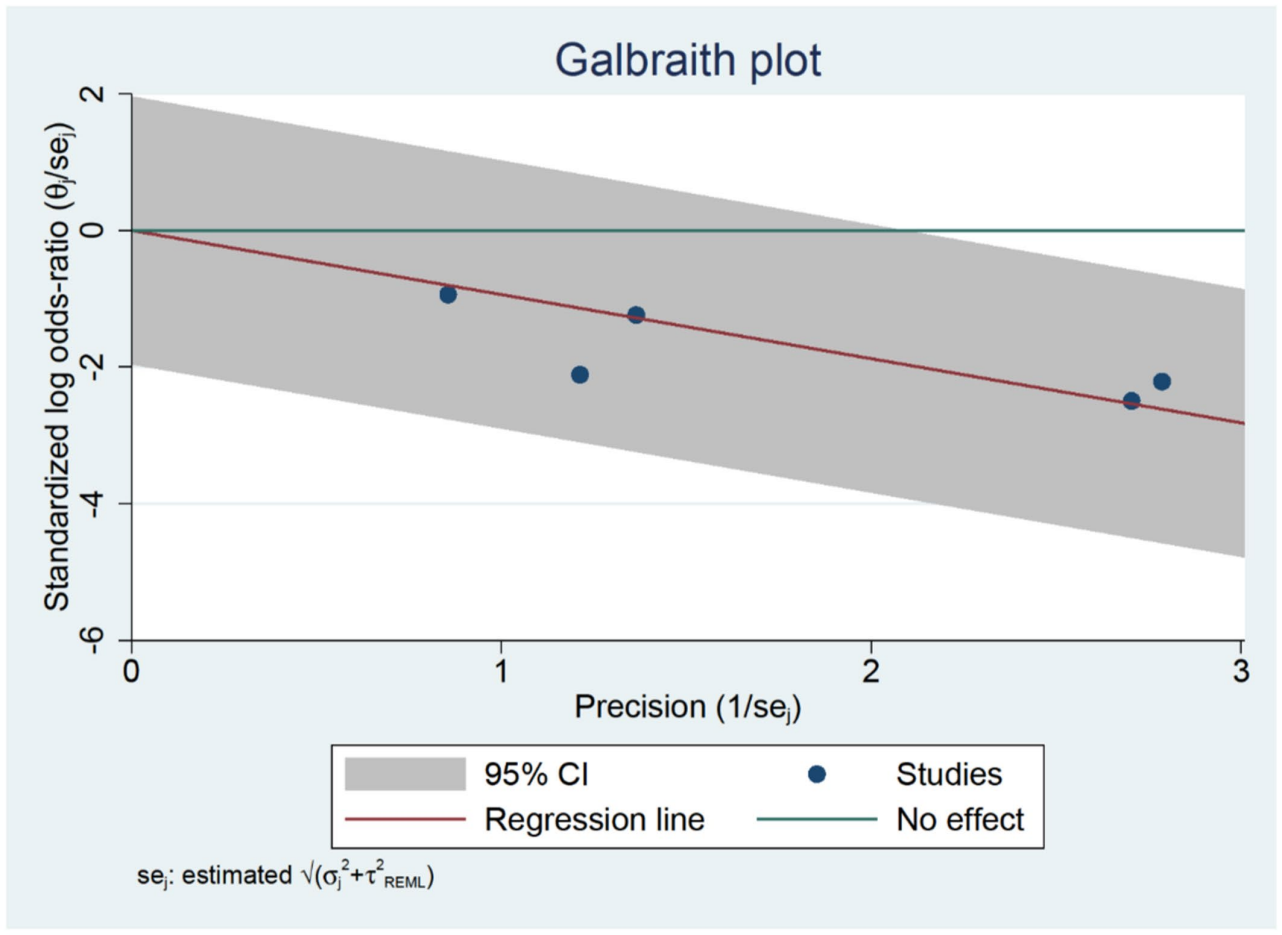
Galbraith plot chart at seven days post-surgery.

**Figure 9:**
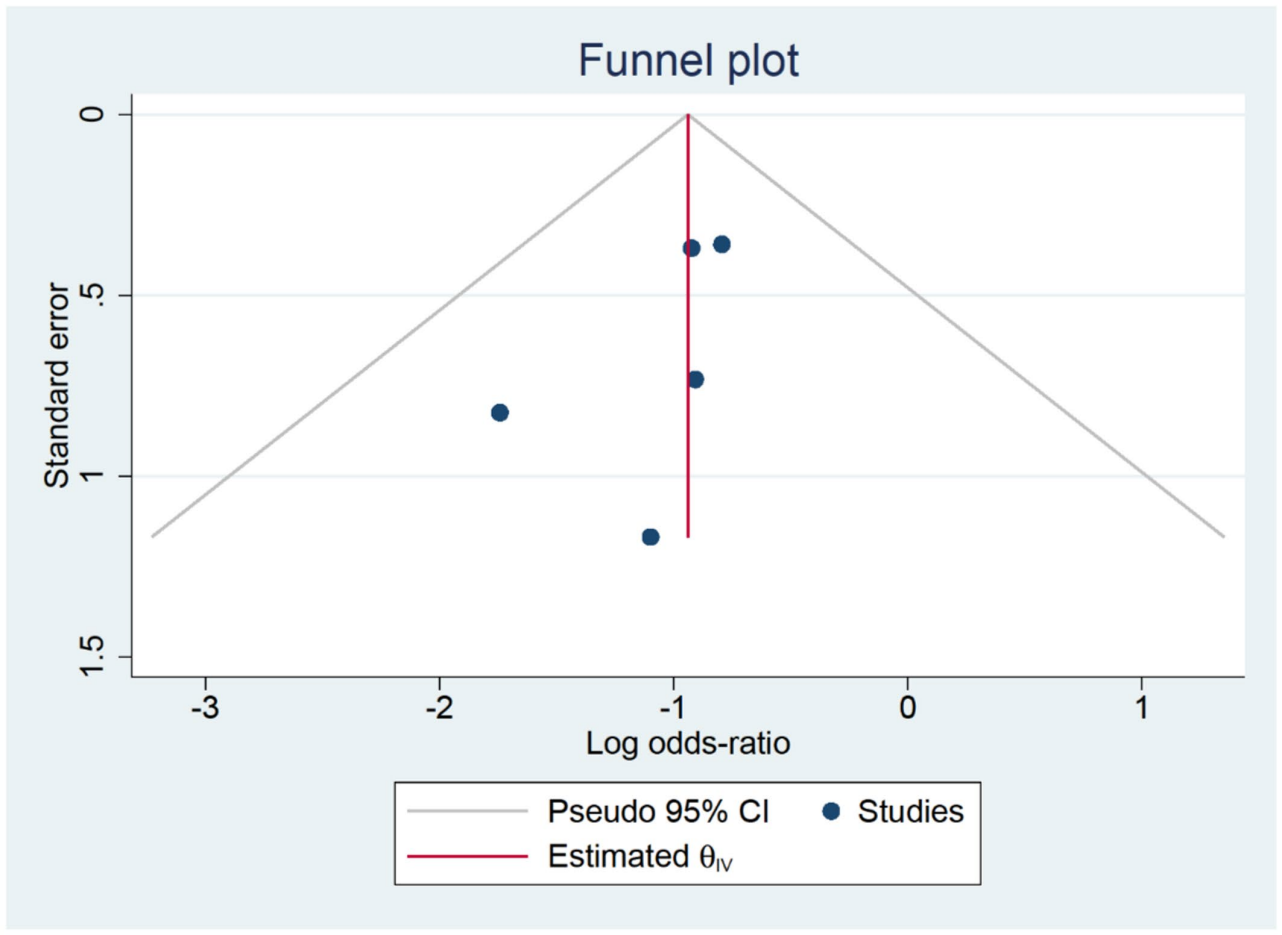
Publication bias funnel plot at seven days post-surgery.

Five RCTs studies (31, 32, 34, 37, 38) were included in our postoperative seven-day delirium analysis. We included the delirium incidence rate closest to the seventh postoperative day. The delirium incidence rate at seven days postoperatively was lower in the TPVB group compared to the control group (log(OR): –0.94, 95% CI [−1.39, −0.49], *p* < 0.001, *I*^2^ = 0.00%) (Figure 7). Heterogeneity assessment using the Galbraith Plot indicated low heterogeneity (Figure 8). We conducted a publication bias funnel plot (Figure 9) and Egger’s test (*p* = 0.247; Supplementary Appendix 3), both of which did not reveal significant evidence of publication bias.

### 3.6 The impact of postoperative pain

**Figure 10:**
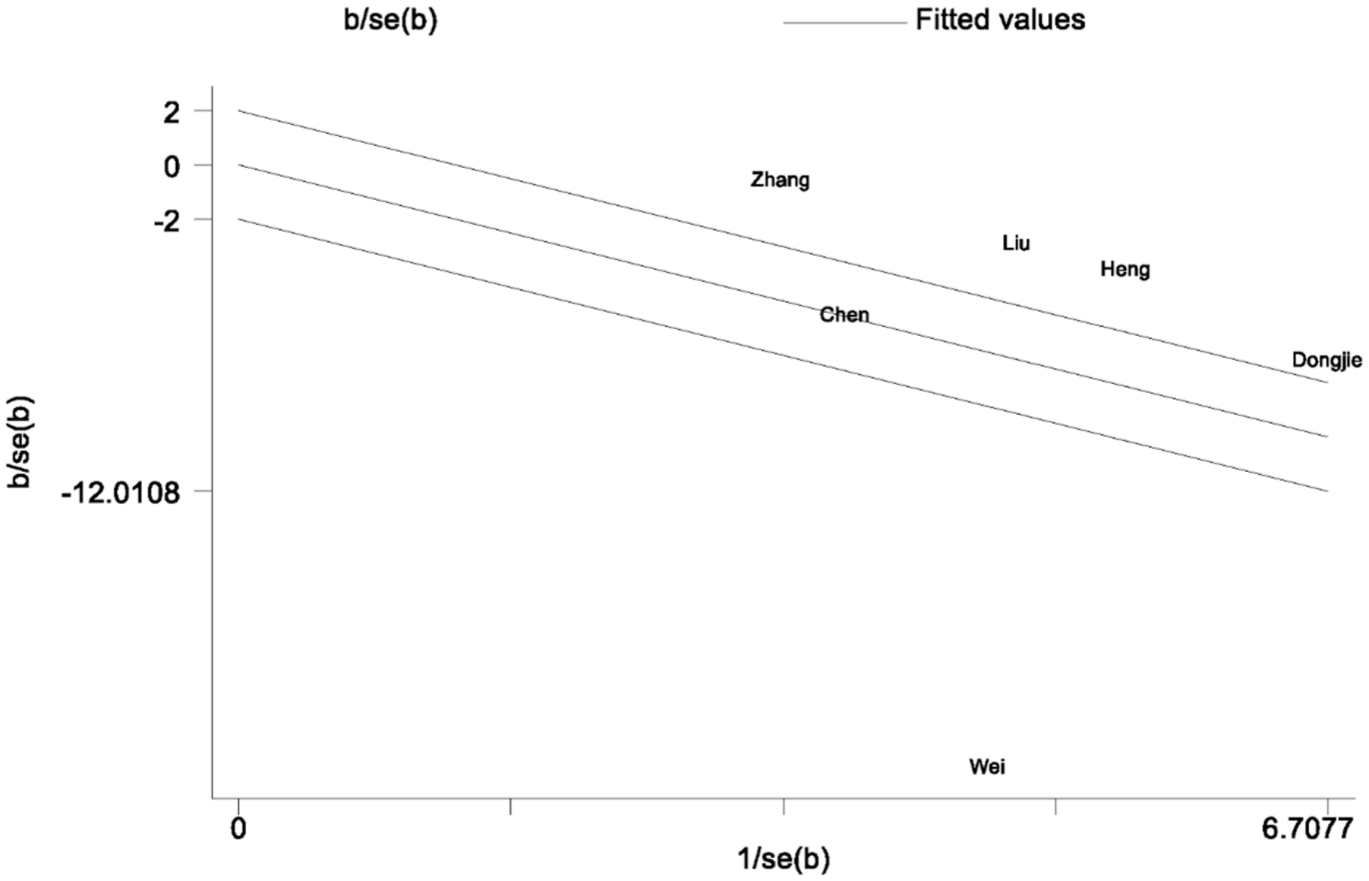
A Galbraith plot related to postoperative pain within a one-day timeframe is presented in the figure.

**Figure 11:**
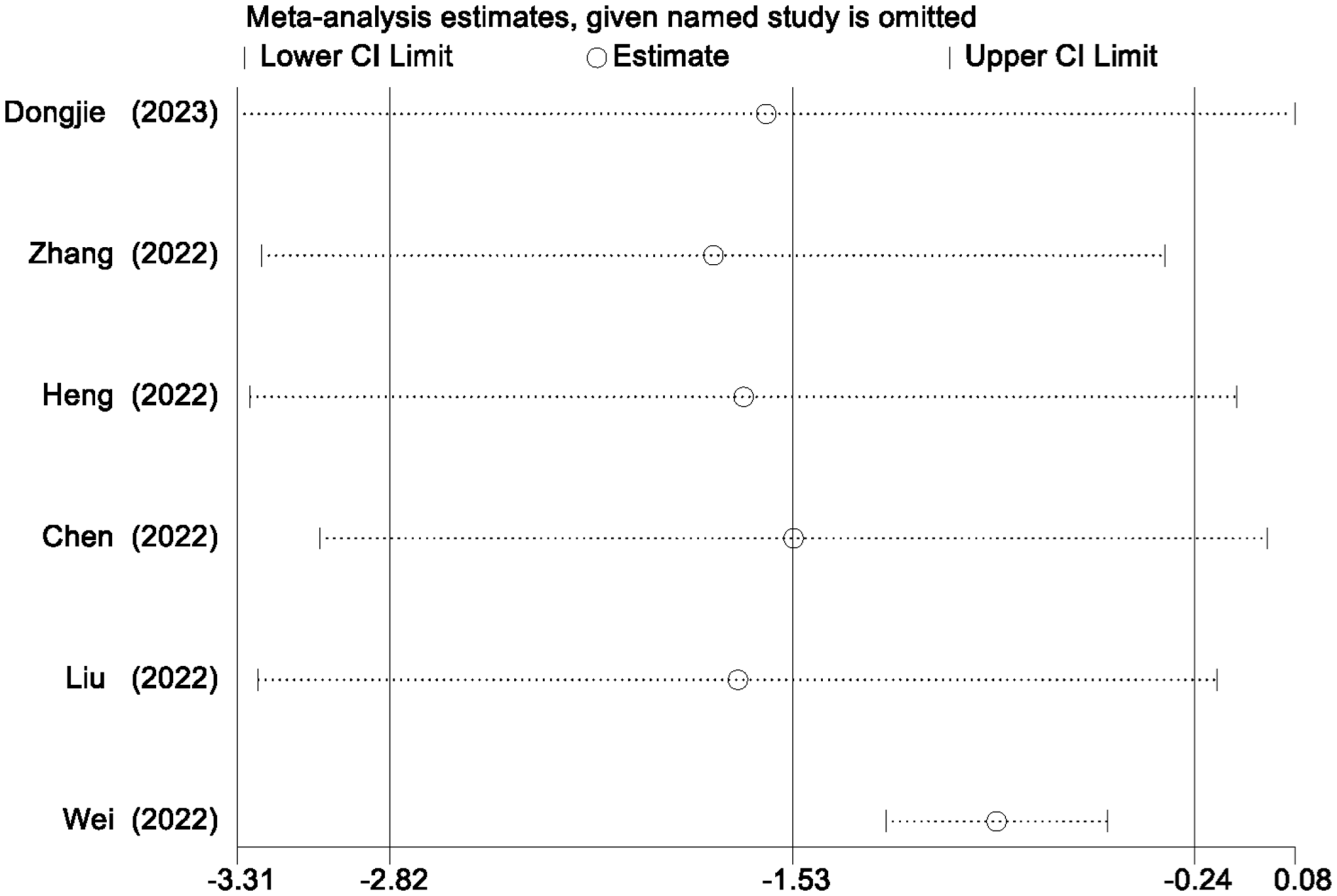
The impact of individual studies on the results.

An analysis of post-surgical first-day pain scores was conducted, omitting a study with a divergent pain assessment approach (39). Six RCTs were integrated into our evaluation (31–36). When performing a meta-analysis on the remaining seven studies that employed VAS pain scores, we observed that on the first day postoperatively, the TPVB group had lower pain scores compared to the control group (*g*: −1.52, 95% CI [−2.87, −0.17], *p* = 0.03, *I*^2^ = 98.50%) (Supplementary Appendix 4). The documented *I*^2^ value signifies a substantial level of heterogeneity. Heterogeneity analysis was conducted using the Galbraith plot (Figure 10), and the influence of individual studies on the results can be observed in Figure 11.

**Figure 12:**
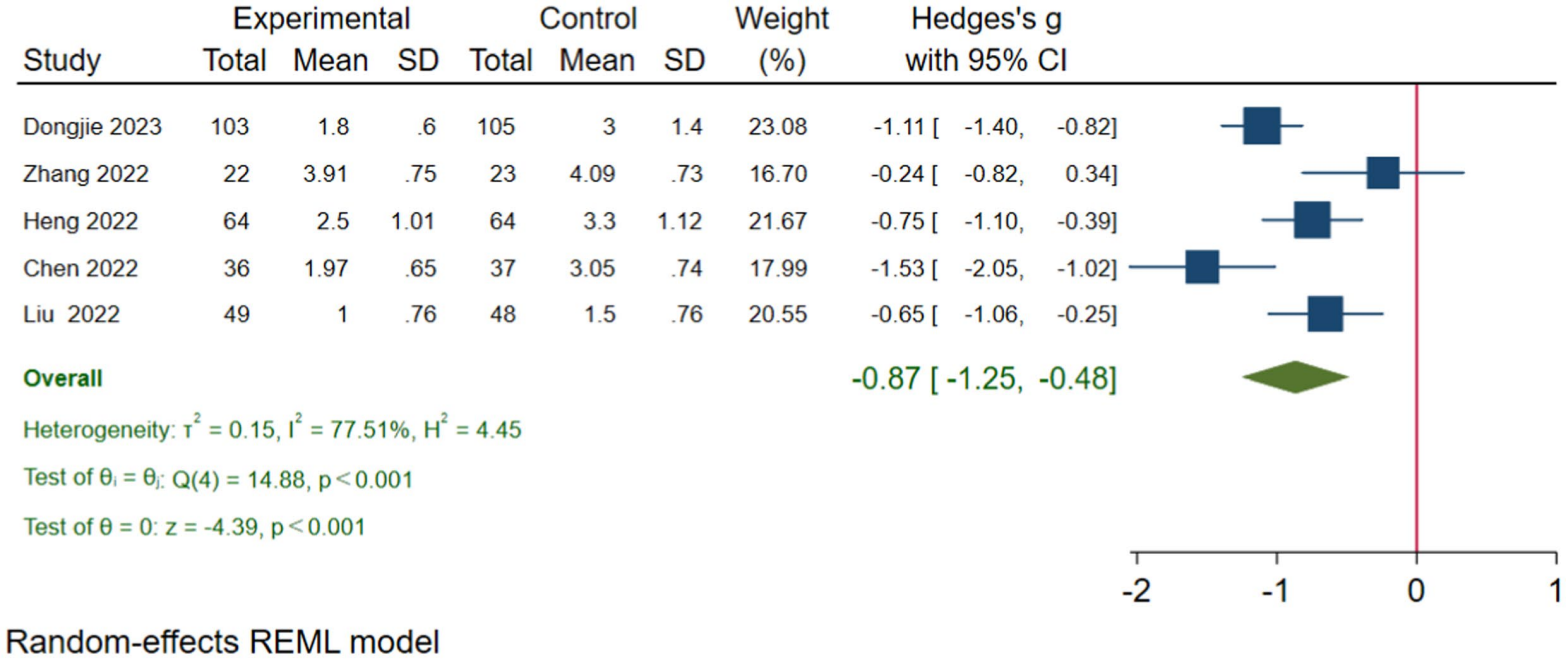
After one day postoperatively, the forest plot of VAS pain scores.

We performed a sensitivity analysis of the pain scores on the first day after surgery. In this process, we excluded the study published by Wei et al. (32) to enhance the reliability and real-world relevance of our study findings. On the first day postoperatively, the VAS pain scores in the TPVB group were significantly lower than those in the control group, and this result was statistically significant (*g*: −0.87, 95% CI [−1.25, −0.48], *p* < 0.001, *I*^2^ = 77.51%) (Figure 12). This suggests that the application of TPVB effectively reduces postoperative pain in patients, leading to improved comfort, enhanced surgical experience, and a smoother recovery process. Nonetheless, it is important to note that there is a relatively high level of heterogeneity among the included studies. Consequently, we should interpret these results with caution. We conducted a publication bias funnel plot and Egger’s test (*p* = 0.662), both of which did not reveal significant evidence of publication bias.

## 4 Discussion

Our systematic review and meta-analysis clearly demonstrate that TPVB can effectively reduce the incidence of delirium in patients after VATS. According to the GRADE methodology, the quality of evidence for this conclusion is rated as high. Additionally, patients receiving TPVB also exhibit reduced postoperative pain scores. The importance of this study lies in indicating the effectiveness of TPVB in reducing the rates of delirium and pain intensity in patients undergoing VATS.

Current perioperative neurocognitive disorders (PND) include delirium (occurring within one week post-surgery or until discharge, whichever happens first), delayed neurocognitive recovery (a decline in cognitive abilities occurring within 30 days post-surgery), and postoperative neurocognitive disorder (lasting up to 12 months) (5). In our investigation, we discerned that TPVB markedly attenuates the incidence of postoperative delirium on the third and seventh days postoperatively, with low heterogeneity. This result suggests that the implementation of TPVB has a short-term impact on the occurrence of PND. However, research on the long-term effects of TPVB on neurocognitive function after the 7th day following VATS is relatively scarce. Therefore, determining the long-term impact of TPVB on neurocognitive function presents a challenge. Further investigation into the effects of TPVB on postoperative neurocognitive function is necessary. These studies should involve larger sample sizes in clinical trials, the use of standardized diagnostic criteria, long-term tracking of neurocognitive function presents after surgery, and comparisons with patients who have not undergone TPVB. Conducting such studies will be instrumental in gaining a more comprehensive understanding of the effects of TPVB on delirium and neurocognitive function. Ultimately, these investigations will furnish more precise recommendations for clinical practice. Through additional research, we can determine whether TPVB can be considered an effective strategy for reducing the occurrence of delirium and improving patients’ neurocognitive function. Furthermore, we can investigate other potential influencing factors to gain a deeper understanding of the mechanisms behind PND and explore preventive measures.

A recent meta-analysis examining the influence of perioperative peripheral nerve blocks on delirium in elderly individuals undergoing hip joint surgery, encompassing 19 randomized controlled trials with a total of 1,977 patients, indicated a decrease in the occurrence of delirium on the third postoperative day (OR: 0.59, 95% CI [0.40–0.87], *p* = 0.007, *I*^2^ = 35%). (40). However, it’s worth noting that another meta-analysis yielded contradictory results, indicating no statistically significant difference in the incidence of postoperative delirium between regional anesthesia and general anesthesia (41). But, this study is subject to considerable heterogeneity influenced by multiple factors. Additionally, there are substantial inconsistencies in both the statistical outcomes and the definitions used. Consequently, drawing a definitive conclusion from this study proves to be challenging.

In our study, we found that TPVB can significantly reduce postoperative pain scores. Postoperative pain is considered one of the significant risk factors for delirium, and this may be associated with the observed decrease in delirium rates on the third postoperative day. At the same time, current research suggests that postoperative opioid use is also one of the risk factors for the occurrence of delirium (42). Considering that TPVB efficiently diminishes postoperative pain levels, it may contribute to a lower delirium rates by minimizing the need for opioid analgesics (43, 44). Nonetheless, it’s worth mentioning that in this study, at the seven-day postoperative time point, patients in the TPVB group also demonstrated a significant decrease in delirium rates. This could be attributed to the ability of TPVB to mitigate perioperative and postoperative stress responses and inflammatory reactions induced by surgery, consequently reducing the incidence of delirium.

In our meta-analysis of pain scores, it is essential to take note that Wei et al. (39) utilized the FLACC scale for pain evaluation, while other studies employed the Visual Analog Scale (VAS) for pain assessment. As a result, we decided to exclude the study conducted by Wei et al. (39) from our analysis. Nevertheless, it is noteworthy that the study conducted by Wei et al. (39) also suggested a tendency towards lower pain scores (OR: 0.55, 95% CI [−0.04, 1.14], *p* = 0.065). Simultaneously, the heterogeneity analysis of pain scores on postoperative day one, as depicted in the Galbraith plot (Figure 10), and the influence of individual studies on the outcomes (Figure 11), clearly demonstrates the substantial heterogeneity evident in the study by Wei et al. published in 2022 (32). This heterogeneity has had a notable impact on the experimental results, potentially introducing inherent bias into the study findings. Consequently, during sensitivity analysis, the aforementioned study was excluded.

Our investigation does face certain limitations. Primarily, due to variations in the timing of delirium diagnosis across the included studies, we extracted delirium incidence rates closest to the third and seventh post-surgical days, incorporating them into the analysis. This approach may inevitably introduce some potential bias. Secondly, multiple diagnostic tools are currently at our disposal for diagnosing delirium, including the Confusion Assessment Method (CAM), the Richmond Agitation-Sedation Scale (RASS), the Memorial Delirium Assessment Scale (MDAS), the Nursing Delirium Screening Scale (Nu-DESC), the Mini-Mental State Examination (MMSE), and the Montreal Cognitive Assessment (MOCA), among others (45, 46). Nonetheless, there’s currently no uniform standard for diagnosing delirium. The research we incorporated utilized different methods for delirium diagnosis, including Nu-DESC, 3D-CAM, MoCA, MMSE, and one study did not explicitly specify its diagnostic approach. These variations in diagnostic methods may potentially influence the study results. Moreover, concerning the implementation of TPVB, most research favored ropivacaine as the primary agent, with a few opted for a combination of lidocaine. Notably, inconsistencies in dosage, volume, concentration, and injection site among these studies. These disparities could potentially affect the effectiveness of TPVB and create variances in the study outcomes.

In summary, this study indicates that TPVB has a positive short-term impact on reducing the incidence of delirium among patients undergoing VATS, with high-quality GRADE evidence supporting this conclusion. However, contradictions remain, and further research is necessary to fully understand TPVB’s effects on neurocognitive function and to refine postoperative management strategies. Such studies are essential for enhancing recovery support for surgical patients and ensuring a more comprehensive approach to their care.

## Data availability statement

The original contributions presented in the study are included in the article/Supplementary material, further inquiries can be directed to the corresponding author.

## Author contributions

XZ: Writing – review & editing, Writing – original draft. WM: Writing – review & editing, Writing – original draft. LZ: Writing – review & editing, Writing – original draft. HZ: Writing – review & editing, Writing – original draft. LC: Writing – review & editing, Writing – original draft. YX: Writing – review & editing, Writing – original draft. LL: Writing – review & editing, Writing – original draft.
