# Supplementary material for: Effect of thoracic paravertebral nerve block on delirium in patients after video-assisted thoracoscopic surgery: a systematic review and meta-analysis of randomized controlled trials
Source: Front Neurol. 2024 Apr 10;15:1347991. doi: 10.3389/fneur.2024.1347991 (PMC11039859; doi:10.3389/fneur.2024.1347991)

Supplementary Material

# Supplementary Data

**Appendix 1**

**Pubmed**

| Search number | Query | Sort By | Filters | Search Details | Results | Time |
| --- | --- | --- | --- | --- | --- | --- |
| 4 | ((((((((((((((((((("Neurocognitive Disorders"[Mesh]) OR (NCD[Title/Abstract])) OR (PND[Title/Abstract])) OR (neurocognitive disorder[Title/Abstract])) OR (perioperative neurocognitive disorders[Title/Abstract])) OR ("Emergence Delirium"[Mesh])) OR ("Delirium "[MESH])) OR ("Postoperative Complications "[MESH])) OR (POD[Title/Abstract])) OR (Delirium[Title/Abstract])) OR (Emergence Delirium[Title/Abstract])) OR (postoperative delirium[Title/Abstract])) OR (delayed neurocognitive recovery[Title/Abstract])) OR ("Postoperative Cognitive Complications"[Mesh])) OR (POCD[Title/Abstract])) OR (postoperative cognitive disorder[Title/Abstract])) OR (postoperative cognitive[Title/Abstract])) OR (postoperative cognitive dysfunction[Title/Abstract])) AND ((((Paravertebral nerve block[Title/Abstract]) OR (Paravertebral block[Title/Abstract])) OR (Thoracic paravertebral nerve block[Title/Abstract])) OR (Thoracic paravertebral block[Title/Abstract]))) AND (((((((((((((((Pulmonary surgery[Title/Abstract]) OR (Pulmonary operation[Title/Abstract])) OR (Pneumonectomies[Title/Abstract])) OR (Endoscopic Lung Volume Reduction[Title/Abstract])) OR (Partial Pneumonectomy[Title/Abstract])) OR (Partial Pneumonectomies[Title/Abstract])) OR (Pneumonectomy, Partial[Title/Abstract])) OR (Bronchoscopic Lung Volume Reduction[Title/Abstract])) OR (Lung Volume Reduction[Title/Abstract])) OR (Reduction, Lung Volume[Title/Abstract])) OR (Volume Reduction, Lung[Title/Abstract])) OR (Lung Volume Reduction Surgery[Title/Abstract])) OR ("Pneumonectomy"[Mesh])) OR (((((((("Pulmonary Surgical Procedures"[Mesh]) OR ("Thoracic Surgery, Video-Assisted"[Mesh])) OR ("Thoracoscopy"[Mesh])) OR ("Thoracotomy"[Mesh])) OR (Chest operation[Title/Abstract])) OR (Pulmonary surgery[Title/Abstract])) OR (Pulmonary operation[Title/Abstract])))) OR (lobectomy[Title/Abstract])) | Publication Date | | ("Neurocognitive Disorders"[MeSH Terms] OR "NCD"[Title/Abstract] OR "PND"[Title/Abstract] OR "neurocognitive disorder"[Title/Abstract] OR "perioperative neurocognitive disorders"[Title/Abstract] OR "Emergence Delirium"[MeSH Terms] OR "Delirium"[MeSH Terms] OR "Postoperative Complications"[MeSH Terms] OR "POD"[Title/Abstract] OR "Delirium"[Title/Abstract] OR "Emergence Delirium"[Title/Abstract] OR "postoperative delirium"[Title/Abstract] OR "delayed neurocognitive recovery"[Title/Abstract] OR "Postoperative Cognitive Complications"[MeSH Terms] OR "POCD"[Title/Abstract] OR "postoperative cognitive disorder"[Title/Abstract] OR "postoperative cognitive"[Title/Abstract] OR "postoperative cognitive dysfunction"[Title/Abstract]) AND ("paravertebral nerve block"[Title/Abstract] OR "paravertebral block"[Title/Abstract] OR "thoracic paravertebral nerve block"[Title/Abstract] OR "thoracic paravertebral block"[Title/Abstract]) AND ("pulmonary surgery"[Title/Abstract] OR "pulmonary operation"[Title/Abstract] OR "Pneumonectomies"[Title/Abstract] OR "endoscopic lung volume reduction"[Title/Abstract] OR "partial pneumonectomy"[Title/Abstract] OR "partial pneumonectomies"[Title/Abstract] OR "pneumonectomy partial"[Title/Abstract] OR "bronchoscopic lung volume reduction"[Title/Abstract] OR "lung volume reduction"[Title/Abstract] OR "reduction lung volume"[Title/Abstract] OR "volume reduction lung"[Title/Abstract] OR "lung volume reduction surgery"[Title/Abstract] OR "Pneumonectomy"[MeSH Terms] OR ("Pulmonary Surgical Procedures"[MeSH Terms] OR "thoracic surgery, video assisted"[MeSH Terms] OR "Thoracoscopy"[MeSH Terms] OR "Thoracotomy"[MeSH Terms] OR "chest operation"[Title/Abstract] OR "pulmonary surgery"[Title/Abstract] OR "pulmonary operation"[Title/Abstract]) OR "lobectomy"[Title/Abstract]) | 170 | 9:24:46 |
| 3 | ((((((((((((((((("Neurocognitive Disorders"[Mesh]) OR (NCD[Title/Abstract])) OR (PND[Title/Abstract])) OR (neurocognitive disorder[Title/Abstract])) OR (perioperative neurocognitive disorders[Title/Abstract])) OR ("Emergence Delirium"[Mesh])) OR ("Delirium "[MESH])) OR ("Postoperative Complications "[MESH])) OR (POD[Title/Abstract])) OR (Delirium[Title/Abstract])) OR (Emergence Delirium[Title/Abstract])) OR (postoperative delirium[Title/Abstract])) OR (delayed neurocognitive recovery[Title/Abstract])) OR ("Postoperative Cognitive Complications"[Mesh])) OR (POCD[Title/Abstract])) OR (postoperative cognitive disorder[Title/Abstract])) OR (postoperative cognitive[Title/Abstract])) OR (postoperative cognitive dysfunction[Title/Abstract]) | Publication Date | | "Neurocognitive Disorders"[MeSH Terms] OR "NCD"[Title/Abstract] OR "PND"[Title/Abstract] OR "neurocognitive disorder"[Title/Abstract] OR "perioperative neurocognitive disorders"[Title/Abstract] OR "Emergence Delirium"[MeSH Terms] OR "Delirium"[MeSH Terms] OR "Postoperative Complications"[MeSH Terms] OR "POD"[Title/Abstract] OR "Delirium"[Title/Abstract] OR "Emergence Delirium"[Title/Abstract] OR "postoperative delirium"[Title/Abstract] OR "delayed neurocognitive recovery"[Title/Abstract] OR "Postoperative Cognitive Complications"[MeSH Terms] OR "POCD"[Title/Abstract] OR "postoperative cognitive disorder"[Title/Abstract] OR "postoperative cognitive"[Title/Abstract] OR "postoperative cognitive dysfunction"[Title/Abstract] | 947,811 | 9:23:47 |
| 2 | (((Paravertebral nerve block[Title/Abstract]) OR (Paravertebral block[Title/Abstract])) OR (Thoracic paravertebral nerve block[Title/Abstract])) OR (Thoracic paravertebral block[Title/Abstract]) | Publication Date | | "paravertebral nerve block"[Title/Abstract] OR "paravertebral block"[Title/Abstract] OR "thoracic paravertebral nerve block"[Title/Abstract] OR "thoracic paravertebral block"[Title/Abstract] | 1,177 | 9:23:33 |
| 1 | ((((((((((((((Pulmonary surgery[Title/Abstract]) OR (Pulmonary operation[Title/Abstract])) OR (Pneumonectomies[Title/Abstract])) OR (Endoscopic Lung Volume Reduction[Title/Abstract])) OR (Partial Pneumonectomy[Title/Abstract])) OR (Partial Pneumonectomies[Title/Abstract])) OR (Pneumonectomy, Partial[Title/Abstract])) OR (Bronchoscopic Lung Volume Reduction[Title/Abstract])) OR (Lung Volume Reduction[Title/Abstract])) OR (Reduction, Lung Volume[Title/Abstract])) OR (Volume Reduction, Lung[Title/Abstract])) OR (Lung Volume Reduction Surgery[Title/Abstract])) OR ("Pneumonectomy"[Mesh])) OR (((((((("Pulmonary Surgical Procedures"[Mesh]) OR ("Thoracic Surgery, Video-Assisted"[Mesh])) OR ("Thoracoscopy"[Mesh])) OR ("Thoracotomy"[Mesh])) OR (Chest operation[Title/Abstract])) OR (Pulmonary surgery[Title/Abstract])) OR (Pulmonary operation[Title/Abstract])))) OR (lobectomy[Title/Abstract]) | Publication Date | | "pulmonary surgery"[Title/Abstract] OR "pulmonary operation"[Title/Abstract] OR "Pneumonectomies"[Title/Abstract] OR "endoscopic lung volume reduction"[Title/Abstract] OR "partial pneumonectomy"[Title/Abstract] OR "partial pneumonectomies"[Title/Abstract] OR "pneumonectomy partial"[Title/Abstract] OR "bronchoscopic lung volume reduction"[Title/Abstract] OR "lung volume reduction"[Title/Abstract] OR "reduction lung volume"[Title/Abstract] OR "volume reduction lung"[Title/Abstract] OR "lung volume reduction surgery"[Title/Abstract] OR "Pneumonectomy"[MeSH Terms] OR "Pulmonary Surgical Procedures"[MeSH Terms] OR "thoracic surgery, video assisted"[MeSH Terms] OR "Thoracoscopy"[MeSH Terms] OR "Thoracotomy"[MeSH Terms] OR "chest operation"[Title/Abstract] OR "pulmonary surgery"[Title/Abstract] OR "pulmonary operation"[Title/Abstract] OR "lobectomy"[Title/Abstract] | 116,608 | 9:23:15 |

**Cochrane**

Search Name:

Date Run: 29/06/2023 18:21:31

Comment:

ID Search Hits

#1 MeSH descriptor: [Neurocognitive Disorders] explode all trees 16626

#2 MeSH descriptor: [Emergence Delirium] explode all trees 211

#3 MeSH descriptor: [Delirium] explode all trees 1395

#4 MeSH descriptor: [Postoperative Complications] explode all trees 49407

#5 MeSH descriptor: [Postoperative Cognitive Complications] explode all trees 67

#6 (postoperative cognitive dysfunction):ti,ab,kw OR (postoperative cognitive disorder):ti,ab,kw OR (POCD):ti,ab,kw OR (delayed neurocognitive recovery):ti,ab,kw OR (postoperative delirium):ti,ab,kw (Word variations have been searched) 3638

#7 (Emergence Delirium):ti,ab,kw OR (Delirium):ti,ab,kw OR (POD):ti,ab,kw OR (perioperative neurocognitive disorder):ti,ab,kw OR (neurocognitive disorder):ti,ab,kw (Word variations have been searched) 10199

#8 (PND):ti,ab,kw OR (NCD):ti,ab,kw (Word variations have been searched) 431

#9 (postoperative cognitive):ti,ab,kw OR (postoperative cognitive disorder):ti,ab,kw OR (postoperative cognitive dysfunction):ti,ab,kw 2772

#10 #1or#2or#3or#4or#5or#6or#7or#8OR#9 74632

#11 (Thoracic paravertebral block):ti,ab,kw OR (Thoracic paravertebral nerve block):ti,ab,kw OR (Paravertebral block):ti,ab,kw OR (Paravertebral nerve block):ti,ab,kw (Word variations have been searched) 1584

#12 MeSH descriptor: [Pulmonary Surgical Procedures] explode all trees 2306

#13 MeSH descriptor: [Thoracic Surgery, Video-Assisted] explode all trees 386

#14 MeSH descriptor: [Thoracoscopy] explode all trees 667

#15 MeSH descriptor: [Thoracotomy] explode all trees 851

#16 MeSH descriptor: [Pneumonectomy] explode all trees 932

#17 MeSH descriptor: [Pneumonectomy] explode all trees 932

#18 MeSH descriptor: [Pneumonectomy] explode all trees 932

#19 (Chest operation):ti,ab,kw OR (Pulmonary surgery):ti,ab,kw OR (Pulmonary operation):ti,ab,kw OR (Pneumonectomies):ti,ab,kw OR (lobectomy):ti,ab,kw 13520

#20 (Endoscopic Lung Volume Reduction):ti,ab,kw OR (Partial Pneumonectomy):ti,ab,kw OR (Partial Pneumonectomies):ti,ab,kw OR (Pneumonectomy, Partial):ti,ab,kw OR (Bronchoscopic Lung Volume Reduction):ti,ab,kw 181

#21 (Lung Volume Reduction):ti,ab,kw OR (Reduction, Lung Volume):ti,ab,kw OR (Volume Reduction, Lung):ti,ab,kw OR (Lung Volume Reduction Surgery):ti,ab,kw 2484

#22 #12or#13or#14or#15or#16or#17or#18or#19or#20or#21 17998

#23 #10and#11and#22 in Trials 149

# Supplementary Figures and Tables

**Appendix 2: The Egger's test for the impact of delirium within three days after surgery.**

Number of studies = 6 Root MSE = .8026

------------------------------------------------------------------------------

Std_Eff | Coefficient Std. err. t P>|t| [95% conf. interval]

-------------+----------------------------------------------------------------

slope | -.4217777 .6500965 -0.65 0.552 -2.226735 1.38318

bias | -.3781952 1.195085 -0.32 0.767 -3.696284 2.939893

------------------------------------------------------------------------------

Test of H0: no small-study effects P = 0.767

**Appendix 3: The Egger's test for the impact of delirium within seven days after surgery.**

Number of studies = 5 Root MSE = .4742

------------------------------------------------------------------------------

Std_Eff | Coefficient Std. err. t P>|t| [95% conf. interval]

-------------+----------------------------------------------------------------

slope | -.5932831 .2644755 -2.24 0.111 -1.434962 .248396

bias | -.741223 .5175038 -1.43 0.247 -2.388151 .9057051

------------------------------------------------------------------------------

Test of H0: no small-study effects P = 0.247

# Appendix 4: Forest plot of the impact on postoperative pain.
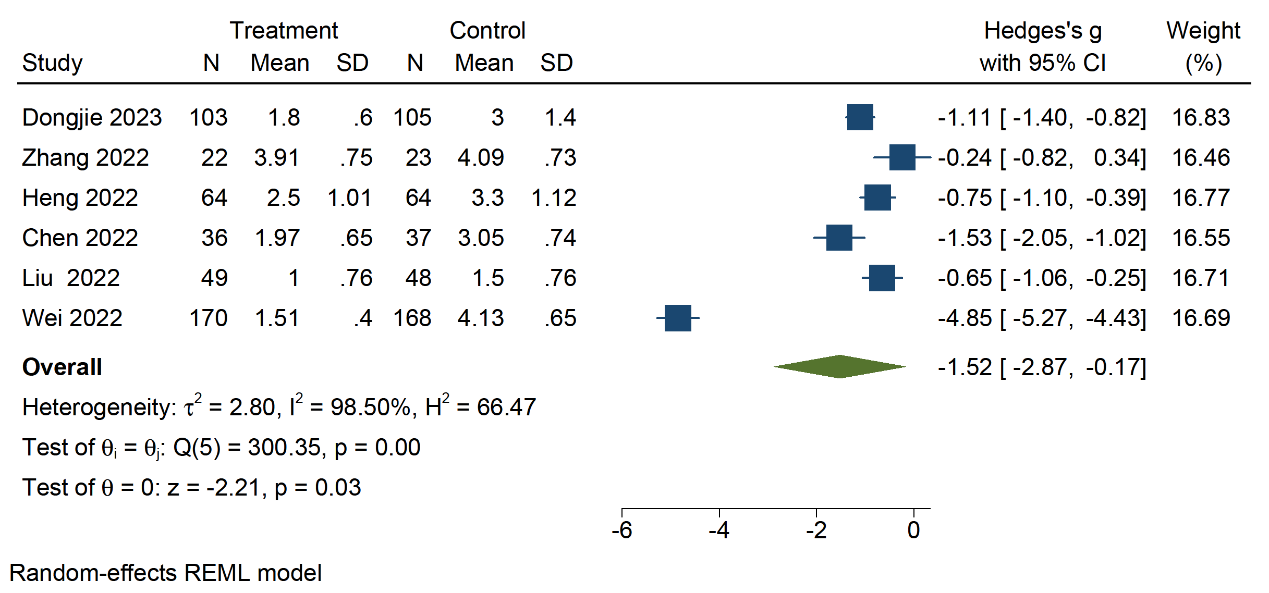


**Appendix 5: Egger's test for the impact on postoperative pain**

Number of studies = 5 Root MSE = 2.131

------------------------------------------------------------------------------

Std_Eff | Coefficient Std. err. t P>|t| [95% conf. interval]

-------------+----------------------------------------------------------------

slope | -1.27794 .7863438 -1.63 0.203 -3.780437 1.224557

bias | 1.884513 3.899196 0.48 0.662 -10.52447 14.2935

------------------------------------------------------------------------------

Test of H0: no small-study effects P = 0.662

**Appendix 6: The impact of postoperative pain: Galbraith Plot Chart**


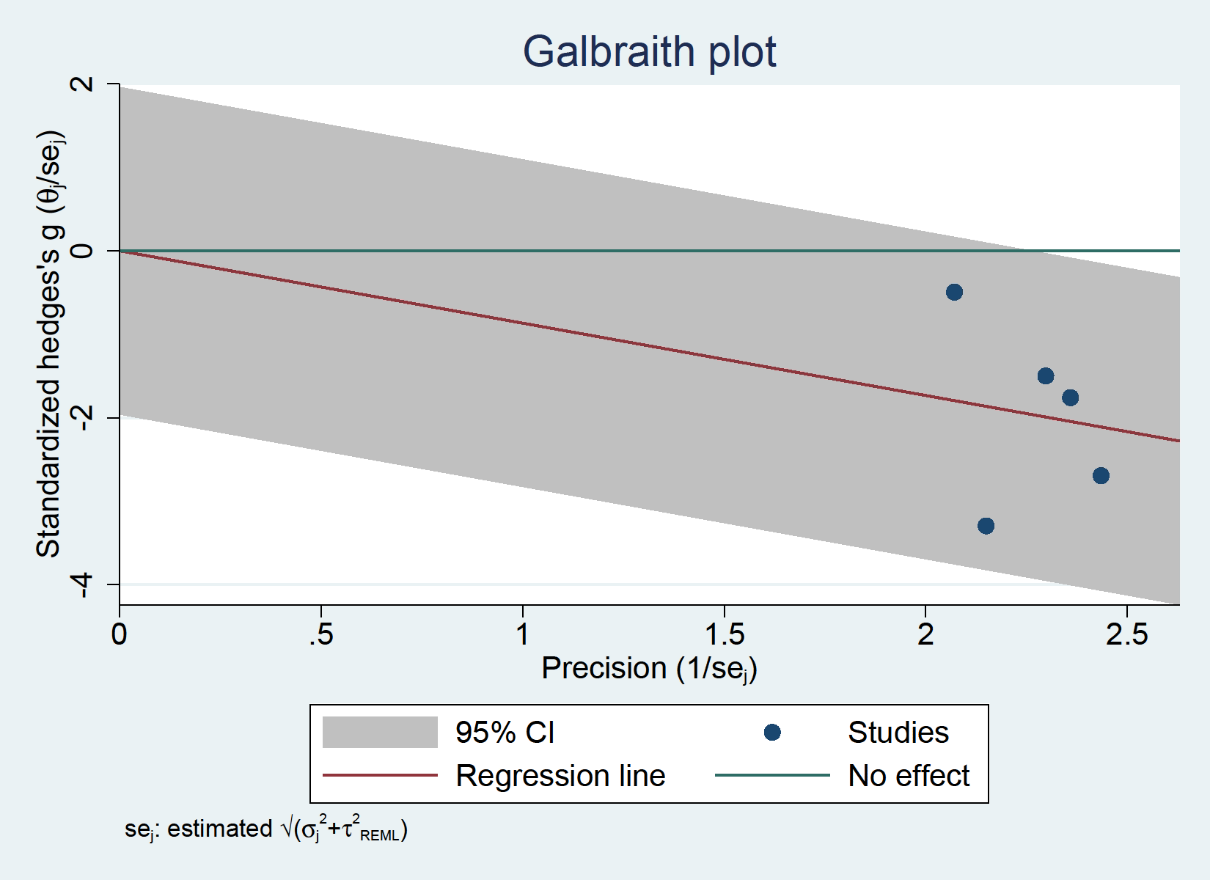

Supplement: Supplementary file 1 [file Data_Sheet_1.docx]
